# Probabilistic Bird Trajectory Forecasting with Heavy-Tailed Uncertainty Modeling for Low-Altitude Airspace Monitoring

**DOI:** 10.3390/s26041270

**Published:** 2026-02-15

**Authors:** Feiyang Song, Zhonghe Liu, Yuyang Zhao, Jingguo Zhu

**Affiliations:** 1 Department of Electrical and Computer Engineering, Northwestern University, Evanston, IL 60208, USA; feiyangsong2027@u.northwestern.edu; 2 Institute of Microelectronics, Chinese Academy of Sciences, Beijing 100029, China; liuzhonghe@ime.ac.cn (Z.L.); zhaoyuyang@ime.ac.cn (Y.Z.); 3 University of Chinese Academy of Sciences, Beijing 100049, China

**Keywords:** bird trajectory forecasting, heavy-tailed uncertainty, airspace monitoring, UAV safety, edge computing

## Abstract

The low-altitude airspace of bird flocks is gradually shared by unmanned aerial vehicles (UAVs), posing safety risks that necessitate accurate trajectory forecasting. However, existing vision-based methods often treat trajectory prediction and UAV detection as separate tasks, assume light-tailed Gaussian noise, and rely on heavy backbones. These limitations, when applied to bird trajectory forecasting, limit uncertainty calibration and embedded deployment in ground-based monocular surveillance. In this work, we propose a unified framework for low-altitude monitoring. Its core, Mini-BirdFormer, combines a lightweight Transformer encoder with a Student-t mixture density head to model heavy-tailed flight dynamics and produce calibrated uncertainty. Experiments on a real-world dataset show the model achieves strong long-horizon performance with only 1.05 million parameters, attaining a minADE of 0.785 m and reducing negative log-likelihood from 1.25 to −2.01 (lower is better) compared with a Gaussian Long Short-Term Memory (LSTM) baseline. Crucially, it enables low-latency inference on resource-constrained platforms at 616 FPS. Additionally, a system-level extension supports zero-shot UAV detection via open-vocabulary learning, attaining 92% recall without false alarms. Results demonstrate that combining heavy-tailed probabilistic modeling with a compact backbone provides a practical, deployable approach for monitoring shared airspace.

## 1. Introduction

The increasing use of low-altitude unmanned aerial vehicles (UAVs) in urban and industrial airspaces has introduced new operational risks, particularly with respect to interactions with avian wildlife [1]. As consumer drones and autonomous inspection systems become more prevalent in airspace shared by bird flocks, the potential for collisions poses a significant threat to both biological conservation and mechanical safety [2,3]. In civil aviation, bird strike mitigation relies on airport-grade radar and acoustic deterrence systems; however, low-altitude UAV operations typically lack access to such advanced sensing infrastructure [1,2]. Ensuring safety in this context therefore requires a vision-based capability that extends beyond passive detection, supporting proactive forecasting of the complex and stochastic trajectories of bird flocks while concurrently monitoring airspace intrusions by other UAVs [4,5].

In the broader field of computer vision, multi-agent trajectory forecasting has achieved remarkable maturity. Foundational data-driven approaches, such as Social-LSTM and Social-GAN [6,7], have successfully modeled human crowd dynamics by capturing social interactions. More recently, Transformer-based architectures [8,9] have been increasingly adopted, leveraging self-attention mechanisms to capture long-range temporal dependencies. To account for the inherent uncertainty in future motion, these models commonly employ probabilistic decoders, most often parameterized as Gaussian Mixture Models (GMMs), to predict multimodal outcomes rather than single deterministic trajectories [10,11].

However, directly applying these pedestrian-centric methods to bird flock monitoring exposes several critical limitations. First, bird motion differs fundamentally from human or vehicular dynamics. Bird flocks exhibit non-rigid collective behaviors marked by rapid accelerations and abrupt directional changes, such as sudden sharp turns or flock splits [12,13]. Such heavy-tailed motion patterns are poorly captured by the light-tailed Gaussian assumptions underlying standard forecasting baselines, which can lead to underestimated risk during sudden maneuvers [11,14]. Second, deployment constraints are particularly restrictive. Edge devices used on UAVs or at remote monitoring stations operate under limited computational budgets, making large-scale Transformer architectures impractical for real-world deployment [15]. Third, visual ambiguity remains a major challenge: birds typically appear as small, deformable targets against cluttered backgrounds, and the scarcity of annotated data severely constrains the training of robust detectors for rare yet safety-critical events such as bird–drone convergence [16,17].

**Figure 1:**
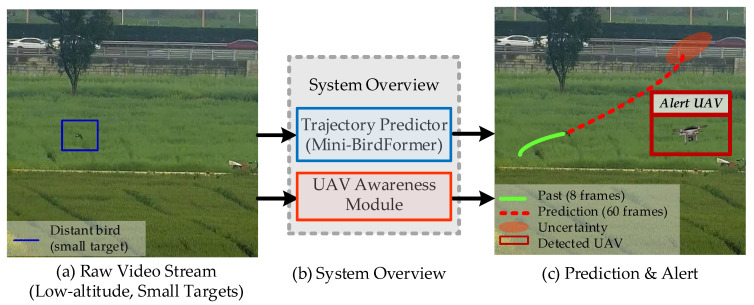
System overview of the proposed airspace monitoring framework. The Mini-BirdFormer predictor forecasts trajectories with uncertainty, while the UAV awareness module (red box in (**c**)) operates in parallel to detect threats without false alarms.

To address these challenges, we present a lightweight probabilistic framework for bird trajectory prediction, augmented by a system-level safety module for airspace monitoring. As illustrated in Figure 1 (where the red box indicates a detected UAV while surrounding birds are correctly ignored), the proposed framework comprises a trajectory forecasting branch and a separate UAV intrusion detection branch, both operating on monocular Red-Green-Blue (RGB) video. In contrast to prior ecological studies that rely on costly 3D reconstruction or Global Positioning System (GPS) telemetry [12,13], the proposed system operates exclusively on monocular RGB video, thereby enabling broader applicability and facilitating practical deployment.

The Mini-BirdFormer trajectory predictor estimates future bird flock trajectories together with associated uncertainty from video-derived observations, while a separate UAV awareness module operates in parallel as a system-level safety extension. The UAV module is not involved in trajectory prediction or model training.

At the core of the trajectory forecasting branch is Mini-BirdFormer, a highly optimized Transformer backbone augmented with a Student-t Mixture Density Network (MDN) head. The Student-t distribution [14], with heavier tails than the Gaussian, offers a more suitable probabilistic prior for modeling the erratic and bursty motion patterns commonly observed in bird flocks. This formulation allows the network to remain robust to tracking noise and abrupt outliers while preserving calibrated uncertainty estimates [11]. To further address data scarcity and computational efficiency, we adopt a deliberately compact architectural design. Ablation studies show that the proposed lightweight architecture, comprising approximately 1.05 million parameters, achieves substantially higher inference speeds than both recurrent and modern vector-based baselines [10]. Moreover, it improves generalization under limited data conditions, thereby mitigating the overfitting effects observed in larger Transformer models.

Finally, to enhance system-level safety without relying on costly labeled data for UAV intrusions, we incorporate a plug-and-play UAV awareness module. By leveraging a pre-trained open-vocabulary vision model (OWL-ViT) in conjunction with a synthetic data generation pipeline, the proposed system can identify potential drone-related risks in a zero-shot manner [16,17]. This additional component provides a complementary layer of situational awareness, enabling unified trajectory uncertainty estimation and airspace risk monitoring within a single forecasting framework.

To systematically present this proposed framework and validate its performance, the remainder of this paper is organized as follows. Section 2 reviews related work on collective bird behavior, trajectory forecasting, and uncertainty modeling, highlighting the limitations of existing approaches. Section 3 details the proposed framework, including the Mini-BirdFormer architecture and the Student-t mixture density formulation. Section 4 presents the experimental setup, quantitative comparisons, and efficiency analysis. Section 5 discusses the architectural trade-offs and domain adaptability. Finally, Section 6 concludes the paper and outlines future research directions.

## 2. Related Work

Prior research relevant to this study spans several closely related domains, including collective bird behavior and airspace safety, trajectory forecasting, uncertainty-aware motion prediction, and UAV detection. Collectively, these lines of work underscore the need for methods that can address the stochastic and safety-critical nature of low-altitude bird–UAV interactions, which remains insufficiently explored in existing approaches.

### 2.1. Collective Bird Behavior and Airspace Safety

The study of collective behavior in bird flocks has a long-established history in ecology and statistical physics [18]. Classical models describe birds as self-propelled agents governed by local interaction rules, such as separation, alignment, and cohesion [12,13]. Although these theories successfully explain the emergence of coherent global patterns, they typically rely on idealized simulations or on precise three-dimensional trajectories obtained from multi-camera capture systems [12,13].

From an airspace safety perspective, prior work has primarily focused on macroscopic bird-strike risk estimation in the vicinity of airports or wind farms [1,3]. Such systems typically rely on radar sensing [1,3], while alternative approaches utilize atmospheric sensors such as pressure and wind data for trajectory reconstruction [19], which restricts their applicability to low-cost, near-ground UAV operations [2]. More critically, these ecological approaches fail to capture the fine-grained, frame-to-frame stochasticity observable in monocular surveillance footage—the sensing modality most commonly available in real-world low-altitude monitoring scenarios. This mismatch motivates the development of lightweight, data-driven forecasting methods that operate directly on video-derived trajectories [3,20].

### 2.2. Trajectory Prediction: From RNNs to Transformers

Trajectory forecasting has reached a high degree of maturity in application domains such as autonomous driving and crowd navigation [4,15]. Early approaches relied on hand-crafted kinematic models and Kalman filtering, which remain effective for approximately linear motion but are unable to capture complex multi-agent interactions [15]. The introduction of recurrent neural networks enabled the modeling of longer temporal dependencies, with Social-LSTM [6] incorporating social pooling mechanisms to encode interactions and Social-GAN [7] employing generative objectives to encourage multimodal predictions.

More recent developments leverage the expressive capacity of Transformer-based architectures. Models such as the approach in [8], AgentFormer [9], and HiVT [10] achieve state-of-the-art performance on pedestrian trajectory benchmarks by jointly modeling temporal and relational structure. However, these methods are primarily optimized for human or vehicular motion, which is constrained by physical rules, social conventions, or road topology. Bird trajectories differ fundamentally from human or vehicular motion. They exhibit rapid accelerations, non-rigid group deformations, and highly nonlinear, heavy-tailed dynamics [12,13,18]. In data-scarce ecological regimes, large Transformer architectures developed for human motion prediction often suffer from overfitting and limited generalization. This observation motivates the development of compact architectures that can generalize effectively under data-scarce conditions.

Recent deep learning approaches have also explored graph-based and reinforcement learning methods for trajectory prediction, particularly in multi-agent settings. Graph-based models explicitly encode interaction structures among agents and have shown strong performance in complex motion scenarios [10,18]. Reinforcement learning–based approaches focus on long-term decision-making under dynamic environments, but often rely on carefully designed reward functions that are difficult to obtain in vision-based bird surveillance scenarios [4].

Trajectory prediction has additionally been studied in the context of UAV navigation and control. Existing UAV-focused methods are typically built upon kinematic models, planning-based frameworks, or reinforcement learning with domain-specific sensors such as GPS or inertial measurements [4]. While effective for controlled flight scenarios, these approaches are not directly applicable to vision-based bird trajectory forecasting. In this work, UAV-related modeling is therefore treated as a complementary downstream task rather than the primary prediction objective.

### 2.3. Uncertainty Modeling in Motion Forecasting

Following the trajectory prediction framework described above, this section introduces the uncertainty modeling component used to capture irregular and multimodal bird motion. Deterministic regression-based approaches, such as those that minimize mean-squared error, tend to produce averaged and physically implausible trajectories in inherently multimodal settings. Consequently, probabilistic modeling has become a central component of modern trajectory forecasting methods. Mixture Density Networks (MDNs), originally formalized by Bishop [3], address this limitation by predicting full probability distributions rather than point estimates and are widely used to represent multiple plausible future outcomes.

Most existing MDN-based approaches adopt Gaussian mixture formulations. However, Gaussian distributions penalize large deviations exponentially. As a result, they are sensitive to outliers and poorly suited for modeling heavy-tailed motion noise [11,14]. Prior work by Kendall and Gal [11] further highlights the importance of explicitly modeling uncertainty in deep vision systems, particularly in safety-critical applications.

However, most existing uncertainty-aware forecasting methods continue to rely on light-tailed Gaussian assumptions, which are insufficient for capturing the heavy-tailed and bursty motion patterns commonly observed in bird flocks.

A summary of these approaches and their limitations in the context of bird flock monitoring is presented in Table 1.

## 3. Methodology

**Figure 2:**
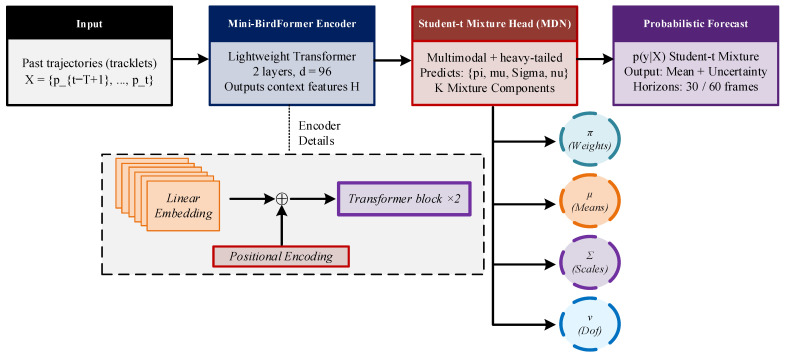
Architecture of the Mini-BirdFormer.

This section describes the proposed framework for probabilistic bird trajectory forecasting, which comprises the Mini-BirdFormer model and a UAV awareness module. Mini-BirdFormer represents the core methodological contribution of this work. Accordingly, Section 3.1, Section 3.2 and Section 3.3 focus exclusively on the Mini-BirdFormer trajectory forecasting model, whereas the UAV awareness module is presented separately in Section 3.4 for completeness. The Mini-BirdFormer architecture, as illustrated in Figure 2, is designed to be lightweight, uncertainty-aware, and suitable for deployment on edge hardware.

The model processes video-derived bird tracklets through a lightweight Transformer encoder coupled with a Student-t mixture density output head to predict future trajectories along with associated uncertainty.

### 3.1. Problem Formulation

Bird-trajectory forecasting is formulated as a probabilistic sequence-to-sequence learning problem. Given a past trajectory segment

(1) $$X_{t}=p_{t-T_{\mathrm{past}}+1},\ldots ,p_{t},p_{t}\in R^{2}$$

the objective is to model the conditional distribution of future positions

(2) $$Y_{t}=p_{t+1},\ldots ,p_{t+T_{\mathrm{fut}}}$$

Instead of predicting a single deterministic path ${\hat{Y}}_{t}$, the model learns the full likelihood $p\;\left(Y_{t}|X_{t};\theta \right)$. This probabilistic formulation is essential for capturing both aleatoric and epistemic uncertainties inherent to biological flight, particularly in the presence of abrupt maneuvers, partial occlusions, and tracking noise [11].

Consistent with common practice in trajectory forecasting, motion is represented in terms of frame-to-frame displacements rather than absolute positions,

(3) $$\Delta p_{\tau }=p_{\tau }-p_{\tau -1}$$

These displacement sequences serve as inputs to the Transformer encoder. Unless otherwise specified, an observation window of $T_{\mathrm{past}}=8$ frames and a prediction horizon of $T_{\mathrm{fut}}=60$ frames (approximately two seconds at 25–30 FPS) are used in all experiments. Future positions are recovered by cumulatively summing the predicted displacements starting from the last observed point.

### 3.2. Trajectory Preprocessing and Encoding

Raw surveillance videos are first processed by a detection–tracking pipeline to extract per-bird tracklets. A lightweight multi-object tracking front-end is adopted, where detections are generated by YOLOX (Megvii Technology, Beijing, China; https://github.com/Megvii-BaseDetection/YOLOX, accessed on 15 February 2025), [21], an anchor-free single-stage detector, and associated across time using ByteTrack [22]. These components were selected based on their demonstrated effectiveness in small-object detection and real-time tracking. Specifically, YOLOX is employed for its efficient anchor-free architecture that enables real-time detection under resource constraints, while ByteTrack’s association logic for low-score detection boxes is crucial for maintaining track continuity of distant birds that frequently appear as low-confidence targets in surveillance footage [22]. After removing short or unreliable tracks, each valid tracklet is segmented into overlapping observation–prediction windows of length $T_{\mathrm{past}}+T_{\mathrm{fut}}$.

Each displacement $\Delta p_{t}$ is projected into a latent feature space of dimension $d_{\mathrm{model}}$ through a linear embedding,

(4) $$e_{t}=W_{\mathrm{in}}\cdot \Delta p_{t}+b_{\mathrm{in}}$$

where $W_{\mathrm{in}}\in R^{d_{\mathrm{model}}\times 2}$ and $b_{\mathrm{in}}\in R^{d_{\mathrm{model}}}$. Throughout this work, $d_{\mathrm{model}}=96$. To preserve temporal order, sinusoidal positional encodings are added to the embedded features.

The resulting sequence $\left\{e_{t}\right\}$ is processed by a compact Transformer encoder composed of two Transformer blocks, each equipped with four attention heads. The hidden dimension of the feed-forward network is set to 192. Each block consists of multi-head self-attention followed by a position-wise feed-forward network, with residual connections and layer normalization applied throughout. Despite its modest capacity of approximately 1.05 million parameters, this lightweight configuration retains the ability to capture long-range temporal dependencies while substantially reducing overfitting compared to deeper and wider Transformer architectures.

Let $H\in R^{T_{\mathrm{past}}\times d_{\mathrm{model}}}$ denote the output sequence from the final encoder layer. A fixed-dimensional representation of the observed history is obtained via temporal average pooling,

(5) $$h_{\mathrm{hist}}=Pool\left(H\right)=\frac{1}{T_{\mathrm{past}}}\sum_{t}H_{t}\in R^{d_{\mathrm{model}}}$$

### 3.3. Probabilistic Output Head: Student-t Mixture Model

Bird motion is inherently multimodal and heavy-tailed: sudden turns, collective accelerations, and tracking jitter often induce large deviations from nominal trajectories. To capture this behavior, a Mixture Density Network (MDN) is adopted, in which the mixture components are modeled using multivariate Student-t distributions rather than Gaussian distributions [11,14]. Unlike Gaussian distributions which decay exponentially, Student-t distributions possess heavier tails, allowing them to assign higher probability to sudden, large-magnitude maneuvers common in bird flight.

Conditioned on the historical representation $h_{\mathrm{hist}}$, the MDN predicts the parameters of a K-component Student-t mixture over future trajectories,

(6) $$p\left(Y|X\right)=\Sigma_{k=1}^{K}\pi_{k}\cdot T\left(y;\mu_{k},\Sigma_{k},\nu_{k}\right)$$

where $\pi_{k}$ are non-negative mixing coefficients satisfying $\sum_{k}\pi_{k}=1$, $\mu_{k}\in R^{2T_{\mathrm{fut}}}$ denote the component means, $\Sigma_{k}$ are block-diagonal scale matrices, and $\nu_{k}$ represent the degrees of freedom. Here, y denotes the vectorized future displacement sequence, and the dimensionality of the distribution is $d=2T_{\mathrm{fut}}$.

The multivariate Student-t density is expressed in factored form as

(7) $$T\left(y;\mu ,\Sigma ,\nu \right)=c\left(\nu ,d,\left|\Sigma \right|\right)\cdot {\left(1+\left(1/\nu \right)\cdot {\left(y-\mu \right)}^{T}\Sigma^{-1}\left(y-\mu \right)\right)}^{-\left(\nu +d\right)/2}$$

where the normalizing constant is given by

(8) $$c\left(\nu ,d,\left|\Sigma \right|\right)=\Gamma \left(\left(\nu +d\right)/2\right)/\left(\Gamma \left(\nu /2\right)\cdot {\left(\nu \pi \right)}^{d/2}\cdot {\left|\Sigma \right|}^{1/2}\right)$$

This heavy-tailed characteristic is well aligned with the empirical motion statistics observed in bird flocks and confers increased robustness to outliers arising from abrupt maneuvers as well as tracking noise [14,19].

In practice, the MDN head is implemented as a shallow multilayer perceptron comprising two hidden layers of width 192 with GELU activations. Mixture weights are normalized via a softmax operation, scale parameters are exponentiated to ensure positivity, and degrees of freedom are passed through a softplus transformation and shifted to remain greater than 2 [14]. Unless otherwise specified, a fixed number of mixture components (*K* = 5) is employed in all experiments for computational efficiency.

Naively maximizing the full mixture likelihood during training often results in mode collapse, whereby only a small subset of mixture components is effectively utilized. To address this, we adopt a Winner-Takes-All (WTA) strategy [23]. In contrast to explicit ensemble formulations that require multiple independent models—thereby linearly increasing computational cost—WTA training encourages the mixture components of a single model to specialize in distinct motion modes [23]. This approach effectively prevents mode collapse while preserving the compact architecture required for high-throughput inference, which would be compromised by the latency overhead of ensemble formulations. For each training sample, the mixture component yielding the highest individual log-likelihood is first identified as:

(9) $$\begin{array}{c} k*=argmax_{k}logT\left(y;\mu_{k},\Sigma_{k},\nu_{k}\right) \end{array}$$

and only this selected component is updated using the corresponding negative log-likelihood:

(10) $$L=-log\pi_{k*}-logT\left(y;\mu_{k*},\Sigma_{k*},\nu_{k*}\right)$$

This training objective encourages different mixture components to specialize in distinct motion modes, thereby fostering diversity within the mixture and stabilizing the optimization of the MDN.

### 3.4. System-Level UAV Awareness Extension (Not Used for Trajectory Prediction)

**Figure 3:**
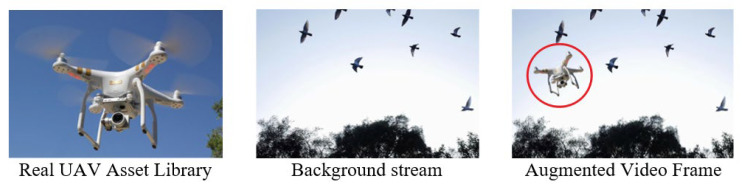
Synthetic UAV Injection Pipeline. The red circle in the augmented video frame highlights the synthetically inserted UAV.

Beyond forecasting bird trajectories, a safety-critical airspace monitoring system must detect unauthorized UAV intrusions. Because genuine bird–UAV encounters are rare and hazardous to collect, we design a synthetic augmentation pipeline (Figure 3) and a zero-shot detector that together provide scalable UAV awareness [24].

UAV templates with transparent backgrounds are composited into real bird-flock surveillance frames along randomized trajectories (encompassing both linear and curved flight paths), enabling controlled evaluation of UAV detection performance without requiring real-world collisions or hazardous data collection.

#### 3.4.1. Synthetic Data Generation

A library of high-resolution UAV templates is first curated in transparent PNG format, covering a diverse range of object geometries and viewing angles. For a subset of bird-only surveillance videos, UAV sprites are synthetically inserted into video frames by randomly sampling the insertion time, scale factor, and two-dimensional flight trajectories, encompassing both linear and curved motion patterns. This controlled compositing procedure produces paired clean and UAV-injected video sequences with precise ground-truth bounding box annotations. Consequently, UAV detection performance can be evaluated in a systematic and fully reproducible manner, while avoiding the practical and safety challenges associated with acquiring real bird–UAV collision data [5].

#### 3.4.2. Zero-Shot Detection Using OWL-ViT

For UAV detection, the OWL-ViT model (Google Research, Mountain View, CA, USA; https://github.com/google-research/scenic, accessed on 15 February 2025) [16,24] is adopted as an open-vocabulary vision–language detector. Given a predefined set of textual prompts [16,17]

T={ “drone”, “UAV”, “quadcopter” },

OWL-ViT produces bounding box predictions and corresponding confidence scores for prompt-aligned objects in each video frame. A frame is classified as unsafe when the maximum UAV-related confidence score exceeds a fixed threshold of δ=0.3.

This modular formulation supports rapid adaptation to previously unseen aerial objects, such as gliders or balloons, through simple modifications to the prompt set T, without requiring task-specific retraining. The detection module integrates naturally with the trajectory prediction pipeline, providing a unified framework for bird–UAV situational awareness.

## 4. Experiments

This section evaluates the proposed framework on the FBD-SV-2024 bird trajectory dataset and a synthetic UAV benchmark. We first describe the experimental setup, including the dataset, evaluation metrics, and implementation details, followed by results on trajectory forecasting accuracy, computational efficiency, robustness to noise, and the performance of the zero-shot UAV awareness module.

### 4.1. Experimental Setup

Experiments are conducted on the FBD-SV-2024 dataset, which provides trajectory annotations extracted from fixed-view bird surveillance videos overlooking agricultural fields and low-altitude landscapes [25]. In total, the dataset comprises 36/12/12 videos and 12,480/4110/4205 trajectories in the training, validation, and test sets, respectively, with average flock sizes of 5.8, 5.6, and 5.9 birds. Each video is recorded at 25–30 Hz with a spatial resolution of 1920 × 1080 and spans a broad range of flock densities, from isolated individuals to dense groups. The dataset is partitioned at the video level into 60% training, 20% validation, and 20% test sets, ensuring that videos captured by the same camera on the same day do not appear across multiple splits.

For evaluation, we follow standard practice in trajectory forecasting and report the Average Displacement Error (ADE) and Final Displacement Error (FDE), measured in meters, for both short-term (30-frame) and long-term (60-frame) prediction horizons. For probabilistic models, ADE and FDE are computed by selecting the predicted trajectory mode that minimizes the corresponding error metric (minADE/minFDE), in accordance with established evaluation protocols for multimodal trajectory forecasting. To assess probabilistic calibration, we additionally report the negative log-likelihood (NLL) of the ground-truth trajectories under the predicted distributions.

To establish a comprehensive performance benchmark, we evaluate the proposed Mini-BirdFormer against four representative baselines spanning deterministic, recurrent, and modern vector-based architectures. Our baseline selection focuses on representative trajectory forecasting paradigms rather than mobile vision backbones. Specifically, LSTM and HiVT are included as widely adopted recurrent and vector-based trajectory models, while the Large Transformer serves as a capacity-matched attention-based baseline operating on the same trajectory input representation. Specifically, we include a deterministic Constant-Velocity (CV) model implemented via a Kalman filter [15], together with a standard LSTM encoder–decoder equipped with a Gaussian density head as a representative recurrent forecasting baseline. To examine the effect of increased model capacity, a larger Transformer variant ($d_{model}$ = 256, 4 layers) coupled with a Student-t mixture head [11,14] is included as a high-capacity baseline, operating on the same trajectory input representation as Mini-BirdFormer. In addition, to benchmark against recent vector-based methods, we incorporate HiVT [10], a hierarchical vector transformer originally developed for autonomous driving. Because HiVT relies on map-based priors in its original formulation, we adapt it to the bird trajectory forecasting setting by removing lane- and map-related inputs and evaluating it under the same single-agent input formulation.

All models share identical input representations, using an observation window of eight frames to predict a sixty-frame horizon. In terms of parameter count, the LSTM, Large Transformer, and HiVT baselines comprise approximately 1.07 M, 5.2 M, and 0.66 M parameters, respectively, compared with 1.05 M for Mini-BirdFormer, with parameter counts reported consistently across all models.

All models are implemented in PyTorch (version 2.7.0; Meta Platforms, Menlo Park, CA, USA) and trained on a single NVIDIA GeForce RTX 4060 GPU (NVIDIA Corporation, Santa Clara, CA, USA). Training is performed using the AdamW optimizer with a base learning rate of 5 × 10^−4^, a weight decay of 0.02, and a batch size of 64. To stabilize optimization on the limited dataset, we adopt a cosine annealing learning rate schedule with a linear warm-up over the first 10% of training epochs and apply dropout with a rate of 0.1 in both attention and feed-forward layers. Training converges in approximately 80 epochs.

Unless stated otherwise, Mini-BirdFormer follows the same architectural and training settings across all experiments. All baseline methods use identical preprocessing pipelines and data splits to ensure a fair and consistent evaluation.

### 4.2. Quantitative Results

#### 4.2.1. Trajectory Forecasting Accuracy

Table 2 summarizes trajectory forecasting performance over 30-frame and 60-frame prediction horizons. To enable a systematic evaluation, we compare Mini-BirdFormer against six baseline configurations spanning deterministic, recurrent, attention-based, and vector-based architectures. In particular, we evaluate the 4-layer Transformer backbone [8] under three different probabilistic output heads—single Gaussian, Gaussian mixture model (GMM, K = 5), and Student-t mixture density network (MDN, K = 5)—to disentangle the effect of the output formulation from that of the backbone architecture.

Several findings emerge. First, the single Gaussian head yields the weakest Transformer performance (ADE@60 = 0.923), confirming that multimodal modeling is necessary for capturing the stochastic dynamics of bird flight. Second, the GMM head achieves the lowest point-prediction error among Transformer variants (ADE@60 = 0.783), indicating that mixture modeling improves trajectory accuracy. Third, the Student-t MDN head achieves improved likelihood-based calibration (NLL = −0.98), suggesting that explicitly modeling heavy-tailed motion uncertainty yields better-calibrated predictive distributions.

Mini-BirdFormer achieves a favorable overall balance across all evaluation dimensions. Despite using only 1.05 M parameters—roughly one-fifth of the large Transformer—it attains an ADE@60 of 0.785, comparable to the GMM variant, while simultaneously achieving the best likelihood-based calibration (NLL = −2.01) and real-time inference at 616 FPS. This improvement in NLL suggests that the compact architecture acts as an implicit regularizer, reducing overfitting under data-scarce ecological conditions and enabling more reliable uncertainty estimates. Note that minADE and minFDE may correspond to different mixture components under multi-modal evaluation, and thus minFDE can be lower than minADE without inconsistency.

HiVT [10] exhibits notably higher errors (ADE@60 = 2.19), reflecting the difficulty of applying vector-based driving models to open-air biological motion forecasting in the absence of structured map priors.

#### 4.2.2. System Efficiency Analysis

Inference speed is measured for the trajectory forecasting module only and does not include preprocessing steps such as object detection and tracking. Experiments are conducted on a laptop equipped with an Intel Core i7-13650HX CPU (Intel Corporation, Santa Clara, CA, USA), 16 GB RAM, running Windows 11. Model efficiency is critical for real-time deployment on resource-constrained platforms such as UAV-mounted modules and remote sensing stations, particularly when trajectory prediction is integrated with tracking and UAV awareness components. Table 3 reports the parameter count and inference speed measured with a batch size of one. Mini-BirdFormer contains only 1.05 M parameters, slightly fewer than the LSTM baseline (1.07 M), yet achieves an inference speed of 616 frames per second on a single GPU, corresponding to approximately 1.62 ms per frame. This throughput is more than twenty times higher than standard video frame rates (30 FPS), leaving sufficient computational margin for additional system modules within an integrated monitoring system.

In addition to efficiency, we evaluate the robustness and long-horizon stability of different models under challenging conditions.

**Figure 4:**
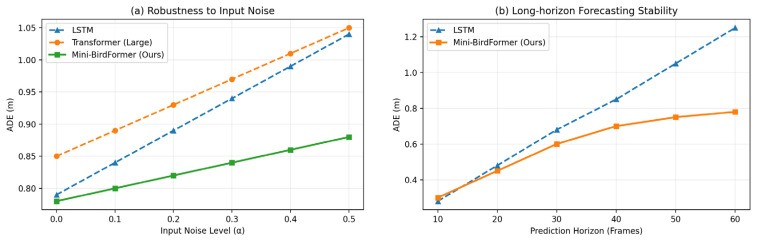
Robustness and long-horizon stability of trajectory forecasting models. Note: The Transformer (Large) baseline is excluded from (**b**) as its error diverges significantly at long horizons.

Figure 4a evaluates robustness to input noise, where zero-mean Gaussian noise with standard deviation α is independently added to each observed past displacement at every timestep during inference [19]. As the noise level increases, the performance of all baselines degrades, while Mini-BirdFormer remains substantially more stable. Under strong perturbations, Mini-BirdFormer achieves approximately 20% lower ADE compared to the Transformer (Large).

Figure 4b examines long-horizon forecasting stability by increasing the prediction horizon from 10 to 60 frames. While the LSTM baseline exhibits rapidly growing prediction errors at longer horizons, Mini-BirdFormer maintains consistently lower ADE, indicating improved temporal stability for long-term trajectory prediction.

**Figure 5:**
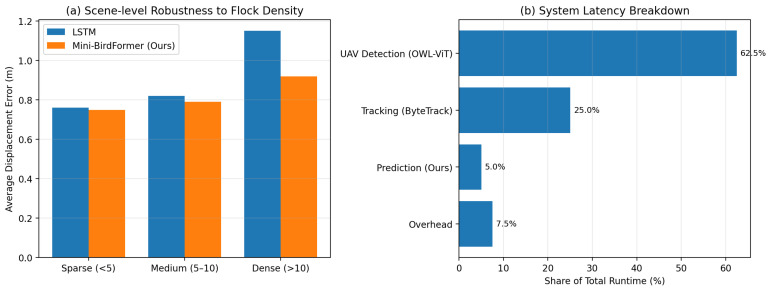
Scene-level robustness and system latency breakdown. (**a**) Scene-level Robustness to Flock Density. (**b**) System Latency Breakdown.

Unlike Figure 4, which evaluates the trajectory forecasting models in isolation, Figure 5 examines scene-level robustness and end-to-end system efficiency when trajectory prediction is integrated with tracking and UAV awareness modules. Figure 5a evaluates scene-level robustness by varying the density of bird flocks, where increasing crowding leads to higher interaction complexity and tracking ambiguity, thereby stressing the full detection–tracking–prediction pipeline and resulting in higher displacement errors due to frequent identity switches and occlusions. To contextualize system-level efficiency beyond the prediction module alone, we further analyze the runtime distribution across different components. As shown in Figure 5b, UAV detection accounts for a larger portion of the overall runtime due to the use of an open-vocabulary vision–language model, whereas the proposed Mini-BirdFormer contributes only a small fraction of the total latency, demonstrating that trajectory forecasting is not the computational bottleneck of the integrated system.

#### 4.2.3. Ablation Study

The results in Table 2 enable a controlled analysis of two orthogonal design axes: probabilistic output formulation and backbone capacity.

Regarding the output head, upgrading from a single Gaussian to a mixture model on the same 4-layer Transformer backbone [8] reduces ADE@60 by 15.2% (from 0.923 to 0.783 for GMM) or 7.4% (to 0.855 for Student-t MDN). These results confirm that mixture-based modeling is essential for multimodal bird trajectories. The Student-t formulation additionally provides improved likelihood-based calibration (NLL = −0.98), reflecting its capacity to model heavy-tailed motion dynamics through adaptive degrees-of-freedom parameters.

Regarding backbone capacity, Mini-BirdFormer (2 layers, 96 dimensions, 1.05 M parameters) achieves an ADE@60 of 0.785 and NLL of −2.01, both improving upon all 5.2 M-parameter Transformer variants. This finding is consistent with the limited effective diversity of bird trajectory data compared to large-scale pedestrian or vehicle datasets [25], where over-parameterized models are prone to overfitting. The compact architecture provides implicit regularization that emphasizes stable, generalizable motion cues.

### 4.3. Safety Extension Evaluation

We evaluate the UAV awareness module on the synthetic benchmark introduced in Section 3.4. Using the prompt set

T={“drone”,“UAV”,“quadcopter”}

the OWL-ViT detector achieves a recall of 92.0% on UAV-injected videos. This high recall is consistently maintained across varying object scales (from distant < 20 px to close-range >100 px), validating the scale-invariance of the open-vocabulary detector. Crucially, on clean bird-only sequences, the detector maintains a precision of 100% (zero false positives), resulting in an F1-score of 0.958, as summarized in Table 3. These results suggest that open-vocabulary detection can function as a reliable safety layer, even in the absence of task-specific training data for bird–UAV interactions.

**Figure 6:**
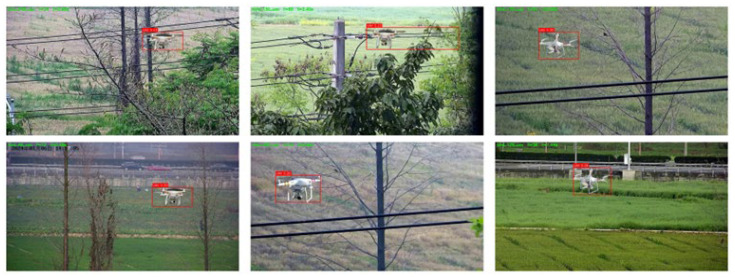
Zero-shot UAV detection results. Example frames illustrating accurate UAV localization (red boxes) across diverse viewpoints and background conditions. The results demonstrate the model’s capability to detect UAVs against complex backgrounds (e.g., trees, fields) without confusion.

Representative qualitative results are presented in Figure 6. The examples demonstrate accurate UAV localization across diverse viewpoints, scales, and background clutter, indicating that the proposed module generalizes effectively to visually challenging surveillance scenarios.

## 5. Discussion

This section discusses the experimental findings, examines key architectural trade-offs, and considers the practical implications and limitations of the proposed framework. The results illustrate how probabilistic modeling and lightweight architectural design jointly support reliable airspace monitoring under constrained computational conditions.

### 5.1. Architectural Efficiency and Domain Adaptability

We observe that the proposed Mini-BirdFormer, despite its limited depth and embedding dimension, achieves lower ADE and FDE than a larger Transformer configuration across all evaluation settings. Although increasing model capacity is often advantageous in large-scale vision and language tasks, this trend does not readily translate to ecological trajectory forecasting.

Bird flight patterns exhibit lower effective diversity than pedestrian or vehicle datasets, characterized by substantial redundancy and sparse coverage of extreme maneuvers [25]. Under such conditions, over-parameterized models—such as the Large Transformer baseline—show degraded generalization, likely due to increased sensitivity to tracking noise and limited motion diversity, an effect further amplified by the inherent jitter present in monocular tracking data. By contrast, our compact architecture implicitly acts as a regularizer, emphasizing the learning of stable and generalizable motion cues that are essential for long-horizon prediction.

The inferior performance of HiVT [10] in map-free bird trajectory settings, particularly for long-horizon prediction (minADE of 2.19 m at 60 frames), underscores the difficulty of directly transferring vector-based driving models to open-air biological motion forecasting. Unlike traffic agents constrained by road topology, bird flocks exhibit stochastic free-flight dynamics in open airspace. Vector-based methods such as HiVT rely heavily on high-definition maps (e.g., lane vectors) to guide prediction. In the absence of these static environmental priors, such models incur substantially higher displacement errors in map-less, open-air scenarios. In contrast, Mini-BirdFormer is specifically designed for map-less environments, enabling robust modeling of heavy-tailed biological motion without imposing topological constraints.

From a system deployment perspective, this efficiency ensures that trajectory forecasting does not constitute a computational bottleneck, thereby facilitating seamless integration into real-time airspace monitoring pipelines.

### 5.2. Heavy-Tailed Uncertainty Modeling with Student-t Distributions

The benefits of Student-t mixture modeling are most clearly reflected in the improved negative log-likelihood and robustness results. Gaussian mixture models, which dominate much of the prior work, assume light-tailed residuals and penalize large deviations exponentially. However, real bird-flight trajectories frequently exhibit abrupt turns, collective accelerations, and transient tracking errors that violate these assumptions.

In contrast, the Student-t distribution assigns non-negligible probability mass to rare yet physically plausible deviations, a property that is reflected in improved NLL and more stable training behavior. The explicitly learned degrees-of-freedom parameter further enables adaptive uncertainty representation under varying levels of motion volatility. These characteristics are consistent with the improved uncertainty calibration observed in the experiments.

### 5.3. Practical Deployment Considerations and Limitations

The compact design of Mini-BirdFormer is well aligned with the computational constraints of low-altitude UAV platforms and remote sensing stations. With only 1.05 million parameters and an inference speed of 616 frames per second, the model provides sufficient computational margin to support real-time trajectory prediction alongside detection and tracking modules.

Several limitations nevertheless remain. Operating exclusively in the image plane precludes explicit depth reasoning and may result in conservative risk estimates under conditions of visual overlap. In addition, the UAV awareness module is evaluated using synthetically composited data, and its reported performance should therefore be interpreted with caution. Finally, the current framework does not explicitly model environmental factors or dense inter-agent interactions, which may further improve long-horizon prediction accuracy.

### 5.4. Relationship to UAV Trajectory Prediction Methods

This section discusses the relationship between the proposed framework and trajectory prediction approaches in the broader UAV and drone research community.

Trajectory prediction within the drone field encompasses diverse tasks, including onboard ego-motion forecasting using GPS/IMU sensor suites [4,15], airspace risk modeling, collision avoidance, and wildlife–UAV interaction studies [2]. The majority of UAV-specific methods operate within known dynamic models and rely on kinematic state estimation—position, velocity, and attitude—for path planning and control. These approaches address a different problem formulation from the one considered in this work.

The present study focuses on third-person, ground-based monocular surveillance, where the observer has no access to agents’ internal states and input consists solely of noisy 2D pixel-coordinate tracklets extracted from video. This setting introduces distinct challenges—including tracking jitter, identity switches, and the absence of depth cues—that motivate the use of visual trajectory forecasting baselines sharing the same input modality [6,8,10].

We acknowledge that incorporating additional comparisons from drone-related trajectory models would further enrich the evaluation. However, the differences in input representation (2D pixel tracklets versus 3D kinematic state vectors) and problem formulation (third-person observation versus ego-motion planning) limit the feasibility of direct numerical comparison under identical experimental conditions. We therefore position Mini-BirdFormer as a complementary component within an integrated airspace monitoring system: the model provides vision-based trajectory forecasting and uncertainty quantification, while the UAV awareness module (Section 3.4) bridges to drone-specific safety applications.

Future work will explore integration with UAV-mounted sensing platforms and multi-modal prediction incorporating both visual observations and kinematic priors, enabling more direct engagement with UAV-specific forecasting methods.

## 6. Conclusions

This paper presented a probabilistic bird trajectory forecasting framework for low-altitude airspace monitoring. The primary contribution was Mini-BirdFormer, a lightweight Transformer-based model equipped with a Student-t mixture density head, designed to capture heavy-tailed motion uncertainty in data-scarce ecological settings. In addition, a UAV awareness module was incorporated as a system-level safety extension to support practical deployment, while remaining independent of the trajectory prediction model and its training. Together, these components enabled efficient and uncertainty-aware monitoring of shared bird–UAV airspace. By combining lightweight temporal modeling with uncertainty-aware prediction, the proposed approach addressed the challenges posed by stochastic bird motion in shared bird–UAV environments.

The core component, Mini-BirdFormer, integrated a compact Transformer encoder with a Student-t Mixture Density Network to model heavy-tailed motion characteristics. Experiments on the FBD-SV-2024 dataset demonstrated improved long-horizon forecasting accuracy and substantially enhanced uncertainty calibration compared with Gaussian-based baselines, while maintaining real-time inference performance at 616 frames per second with only 1.05 million parameters.

In addition, a zero-shot UAV awareness module based on open-vocabulary detection provided complementary safety monitoring without requiring annotated bird–UAV interaction data. Future work will extend the framework toward three-dimensional trajectory reasoning, interaction-aware modeling, and validation under real-world bird–UAV coexistence scenarios.

## Figures and Tables

**Table 1:** Comparison of trajectory forecasting approaches and their limitations.

Approach	Domain	Model Type	Uncertainty	Key Limitations for Bird Monitoring
Kalman Filter [15]	General	Linear/Kinematic	Gaussian	Fails to capture non-linear social behaviors.
Social-LSTM [6]	Pedestrian	RNN	Gaussian	Long-term error accumulation; underestimates risk.
Transformer [8]	Auto-driving	Attention	Gaussian/GMM	Over-parameterized; prone to overfitting on small data.
HiVT [10]	Auto-driving	Vector-based	Laplace	Relies on map priors (lanes); high inference latency.
Mini-BirdFormer	Bird Flock	Lightweight	Student-t	(Ours) Optimized for heavy-tailed motion & edge usage.

**Table 2:** Trajectory Forecasting Performance on the FBD-SV-2024 Test Set. ↓ indicates lower is better. – denotes not applicable.

Model	Backbone	Head	minADE (30)	minFDE (30)	minADE (60)	minFDE (60)	NLL ↓	Params (M)	FPS
CV (Kalman)	Kalman	Deterministic	0.958	1.012	1.120	1.350	–	0	–
LSTM [6]	RNN	Gaussian	0.789	0.786	0.787	0.784	+1.25	1.07	–
Transformer [8]	4L–256d	Gaussian	0.959	0.886	0.923	0.959	–	5.2 M	–
Transformer [8]	4L–256d	GMM (K = 5)	0.784	0.784	0.783	0.785	–	5.2 M	–
Transformer (Large) [8]	4L–256d	Student-t	0.861	0.820	0.855	0.877	–0.98	5.2	–
HiVT (CVPR’22) [10]	Vector	Laplace	1.69	1.57	2.19	1.87	1.50	0.66	–
Mini-BirdFormer (Ours)	2L–96d	Student-t	0.790	0.785	0.785	0.780	–2.01	1.05	616

**Table 3:** System Efficiency and UAV Detection Performance.

Module	Metric	Value	Unit	Notes
*Trajectory Predictor (Mini-BirdFormer)*				
Model Size		1.05	M	Smaller than LSTM
Inference Speed		616	FPS	Prediction module only
Latency		1.62	ms	Per frame (prediction)
*Safety Extension (UAV Detection)*				
Recall		92.0	%	Synthetic UAV benchmark
False Alarm		0.0	%	Clean bird videos

## Data Availability

The FBD-SV-2024 dataset used in this study is publicly available in [25].
